# Trends in Private Equity Consolidation in Cardiovascular Care

**DOI:** 10.1001/jamahealthforum.2024.1478

**Published:** 2024-06-14

**Authors:** Yashaswini Singh, Megha Reddy, Christopher Whaley

**Affiliations:** 1 Department of Health Services, Policy, and Practice, Brown University School of Public Health, Providence, Rhode Island

## Abstract

This cross-sectional study examines the growth in numbers and geographic locations of private equity acquisitions in cardiology across the US.

## Introduction

The US health care system has witnessed a rapid increase in the consolidation of physician practices by private equity (PE) firms. Cardiology is a recent area of PE growth, partly due to the inclusion of several cardiovascular procedures reimbursed by the Centers for Medicare & Medicaid Services in ambulatory surgery settings, fragmentation of cardiology practices, and rising prevalence and severity of cardiac disease.^1^ In general, PE acquisitions follow a platform and add-on strategy to consolidate multiple small practices in the same geographic market into a single-platform practice.^2^ As a result, some markets are more affected than others.^3^ The absence of systematic evidence on PE acquisitions limits the ability of policymakers, clinicians, and researchers to appropriately monitor or regulate PE growth. We examined the number of acquisitions and affiliated locations and geographic concentration of PE-acquired cardiology platforms, starting from the first known acquisition in 2019.

## Methods

This cross-sectional study used data from PitchBook^4^ on PE acquisitions of cardiology practices from January 1, 2019, to December 31, 2023. We manually verified and expanded this list in January 2024, using publicly available data, press releases, and clinic websites. This study was approved by Brown University institutional review board with a waiver of informed consent as data do not include individual information. The study follows the STROBE reporting guideline.

We calculated the percentage of cardiology practices affiliated with PE acquisition in each state. The denominator represents the approximate number and location of cardiology practices in each state, identified using Medicare Care Compare^5^ (additional detail provided in the eMethods in Supplement 1). Tableau Desktop, version 2024.1.2 (Salesforce, Inc) was used for the data analysis.

## Results

**Figure 1.**
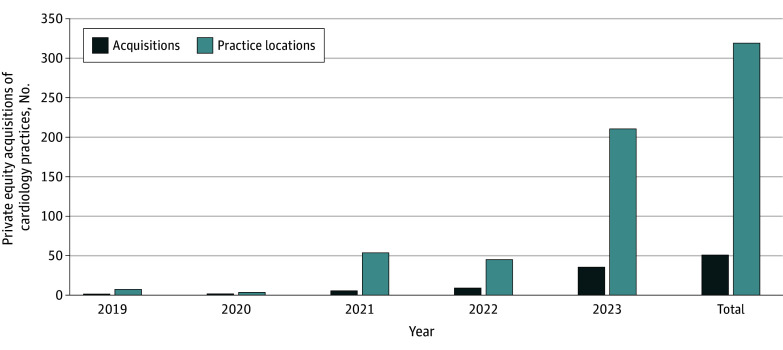
Growth in Private Equity Acquisitions in Cardiology, 2019-2023

Private equity acquisitions of cardiology practices increased from 1 with 7 locations in 2019 to 50 with 320 locations as of 2023 (Figure 1). Collectively, these acquisitions represent 3.9% (332 of 8223) of cardiology practices in the US. Acquisitions in 2023 account for 70.0 (35 of 50) of all identified acquisitions to date.

**Figure 2.**
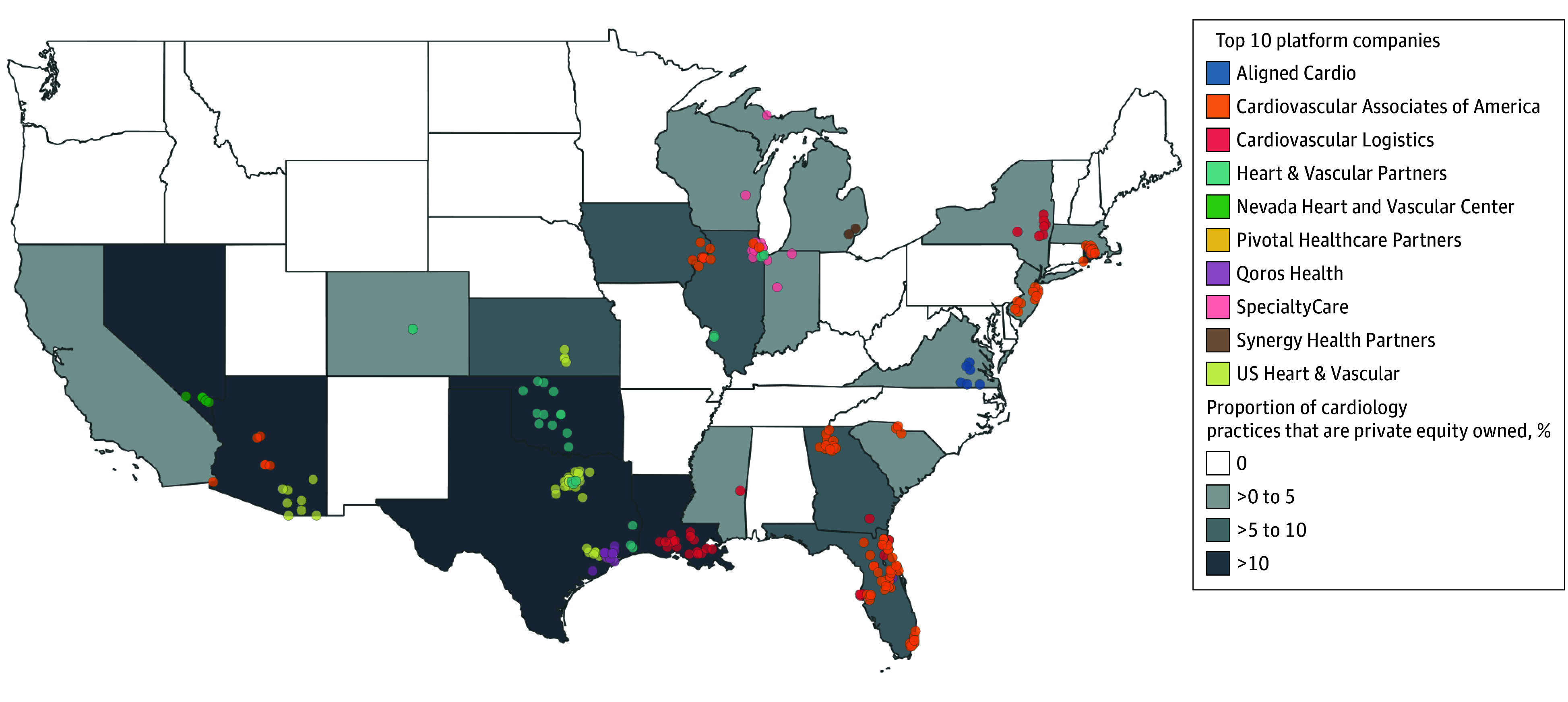
Geographic Distribution of Private Equity–Backed Cardiology Platforms, 2019-2023 The top 10 cardiology platforms represent those with the largest number of private equity–acquired locations as of 2023.

Geographic variation in PE-acquired cardiology platforms spans 22 states, with the largest number of locations in Florida (80), Texas (76), and Arizona (29) (Figure 2). Private equity–acquired cardiology practices accounted for more than 10% of cardiology practices in 7 states, including Rhode Island (13 of 35 [37.1%]), Nevada (14 of 53 [26.4%]), Louisiana (21 of 89 [23.6%]), Arizona (29 of 165 [17.6%]), Oklahoma (13 of 82 [15.9%]), Texas (76 of 675 [11.3%]), and Florida (80 of 798 [10.0%]).

Twenty-nine states and the District of Columbia had no identified acquisitions. The largest PE-acquired platforms are Cardiovascular Associates of America (108 locations in 9 states) and US Heart & Vascular (78 acquisitions in 3 states). The 2 largest cardiology platforms operate all PE-acquired practices in Arizona, Georgia, Iowa, Kansas, New Jersey, Rhode Island, and South Carolina and more than 60% of acquired practices in Florida, Georgia, and Texas.

## Discussion

These findings show that PE acquisitions in cardiology have increased rapidly, with 2023 acquisitions accounting for 70.0% of acquisitions since 2019. Texas, Florida, Arizona, and Nevada have a large presence of PE-acquired cardiology practices, consistent with prior research.^3^ Many cardiology platforms have a regional focus and have expanded their geographic footprint through consolidation of multiple physician practices in a given market.

A key area for future research is the cumulative influence of add-on consolidation by PE funds on competition, which might lead to higher prices with implications for quality and access. As PE acquisitions in cardiology unfold, monitoring outcomes for the clinical workforce, whether practices undergo secondary buyouts,^6^ and the regulatory and market factors that may make certain geographies attractive targets for PE is important.

 A study limitation is that the data may not have captured all PE acquisitions. Secondary data from Medicare Care Compare group physicians into practices by location and may differ from alternate approaches to identifying practice affiliations, such as tax identification numbers in the Medicare Data on Provider Practice and Specialty.

## Notes

Supplement 1.
eMethods.

eReferences

Supplement 2.
Data Sharing Statement
