# Supplementary material for: Trends in Private Equity Consolidation in Cardiovascular Care
Source: JAMA Health Forum. 2024 Jun 14;5(6):e241478. doi: 10.1001/jamahealthforum.2024.1478 (PMC11179124; doi:10.1001/jamahealthforum.2024.1478)
Supplement: Supplement 1. — eMethods. eReferences [file jamahealthforum-e241478-s001.pdf]

Supplemental Online Content

Singh Y, Reddy M, Whaley C. Trends in private equity consolidation in cardiovascular care. *JAMA Health Forum*. 2024;5(6):e241478. doi:10.1001/jamahealthforum.2024.1478

eMethods.

eReferences

This supplemental material has been provided by the authors to give readers additional information about their work.

© 2024 Singh Y et al. *JAMA Health Forum.*

eMethods

Identifying PE acquisitions

Our primary source of data on private equity (PE) transactions is a proprietary list of deals in the "Clinics and outpatient services" sector compiled by Pitchbook Inc., a financial database that tracks mergers and acquisitions across industries and has been used by other studies examining PE in health care.[1–3]

Information on PE acquisition of cardiology practices from 2019 to 2023 was derived from Pitchbook. Acquisition data from Pitchbook included the name and a description of the acquired practice, city and state of the acquired locations, a description of the deal, and the announcement date of the deal.

We supplement the Pitchbook data in two ways. First, we conducted additional Internet searches that yielded an additional number of acquisitions not reported by Pitchbook. Second, we manually verified and expanded our list of acquisitions using a combination of press releases, industry reports, and physician practice websites. This process allows us to identify standalone practice sites associated with each practice acquisition, as well as to account for changes in practice names.

Identifying the number of cardiology practices in each state

To identify the total number of cardiology practices in the US (denominator in Figure 2), we used the 2023 Doctors and Clinicians National Downloadable File available online through the Medicare Care Compare Database. This dataset tracks individual level data on clinicians and their affiliated practice locations and can be downloaded from the Centers for Medicare and Medicaid Services website.[4]

To identify cardiology practices, we examined unique practice locations in the Medicare Care Compare database that had at least one cardiologist. For the purpose of our analysis, we defined a cardiologist as a clinician with an MD or DO credential whose primary specialty was described as Advanced Heart Failure, Cardiac Electrophysiology, Cardiac Surgery, Cardiovascular Disease, or Interventional Cardiology.

This process generated 8223 unique cardiology practice locations across the US, consistent with the number of practices identified in previous research.[5] To arrive at the denominator for our analysis, we calculated the total number (sum) of the number of cardiology locations in each state, using the practice address and state information available in the Medicare Care Compare database.

© 2024 Singh Y et al. *JAMA Health Forum.*

eReferences

1. Singh Y, Song Z, Polsky D, Bruch JD, Zhu JM. Association of Private Equity Acquisition of Physician Practices With Changes in Health Care Spending and Utilization. *JAMA Health Forum*. 2022;3(9).

2. Braun RT, Bond AM, Qian Y, Zhang M, Casalino LP. Private Equity In Dermatology: Effect On Price, Utilization, And Spending | Health Affairs. Published 2021. Accessed August 21, 2023. https://www.healthaffairs.org/doi/full/10.1377/hlthaff.2020.02062?casa_token=3WFaIzKVh18AAAAA%3ANk1dkpgZTIDkTV9prRqH8HiPGoe2O41x7uAmhs4w7Eh8oOzJbHZbxdxANEda-K52dSr8qZN3vQ

3. Forgia AL, Bond AM, Braun RT, et al. Association of physician management companies and private equity investment with commercial health care prices paid to anesthesia practitioners. *JAMA Intern Med*. 2022;182(4):396-404.

4. Doctors and clinicians data archive | Provider Data Catalog. Accessed February 23, 2024. https://data.cms.gov/provider-data/archived-data/doctors-clinicians

5. Figueroa JF, Lam MB, Orav EJ, Joynt Maddox KE. Consolidation Among Cardiologists Across U.S. Practices Over Time. *J Am Coll Cardiol*. 2020;76(5):590-593. doi:10.1016/j.jacc.2020.04.081

© 2024 Singh Y et al. *JAMA Health Forum.*
