# Supplementary material for: Trends in Private Equity Consolidation in Cardiovascular Care
Source: JAMA Health Forum. 2024 Jun 14;5(6):e241478. doi: 10.1001/jamahealthforum.2024.1478 (PMC11179124; doi:10.1001/jamahealthforum.2024.1478)
Supplement: Supplement 2. — Data Sharing Statement [file jamahealthforum-e241478-s002.pdf]

Data Sharing Statement

Singh. Trends in Private Equity Consolidation in Cardiovascular Care. *JAMA Health Forum*. Published June 14, 2024. doi:10.1001/jamahealthforum.2024.1478

Data
Data available: No
